# Poor outcomes in both infection and colonization with carbapenem-resistant Enterobacterales

**DOI:** 10.1017/ice.2022.4

**Published:** 2022-12

**Authors:** Jessica R. Howard-Anderson, Michelle Earley, Lauren Komarow, Lilian Abbo, Deverick J. Anderson, Jason C. Gallagher, Matthew Grant, Angela Kim, Robert A. Bonomo, David van Duin, L. Silvia Muñoz-Price, Jesse T. Jacob

**Affiliations:** 1 Division of Infectious Diseases, Department of Medicine, Emory University School of Medicine, Atlanta, Georgia; 2 The Biostatistics Center, The George Washington University, Rockville, Maryland; 3 Division of Infectious Diseases, University of Miami Miller School of Medicine and Jackson Health System, Miami, Florida; 4 Duke Center for Antimicrobial Stewardship and Infection Prevention, Duke University, Durham, North Carolina; 5 Department of Pharmacy Practice, Temple University, Philadelphia, Pennsylvania; 6 Section of Infectious Diseases, Department of Internal Medicine, Yale School of Medicine, New Haven, Connecticut; 7 Division of Infectious Diseases, Northwell Health, Manhasset, New York; 8 Louis Stokes Cleveland Department of Veterans’ Affairs Medical, Center, Cleveland, Ohio; 9 Case Western Reserve University–Cleveland VAMC Center for Antimicrobial Resistance and Epidemiology (Case VA CARES), Cleveland, Ohio; 10 Departments of Pharmacology, Molecular Biology and Microbiology, Biochemistry, and Proteomics and Bioinformatics, Case Western Reserve University School of Medicine, Cleveland, Ohio; 11 Department of Medicine, Case Western Reserve University School of Medicine, Cleveland, Ohio; 12 Division of Infectious Diseases, University of North Carolina, Chapel Hill, North Carolina; 13 Division of Infectious Diseases, Department of Medicine, Medical College of Wisconsin, Milwaukee, Wisconsin

## Abstract

**Objectives:**

To describe the epidemiology of patients with nonintestinal carbapenem-resistant Enterobacterales (CRE) colonization and to compare clinical outcomes of these patients to those with CRE infection.

**Design:**

A secondary analysis of Consortium on Resistance Against Carbapenems in *Klebsiella* and other Enterobacteriaceae 2 (CRACKLE-2), a prospective observational cohort.

**Setting:**

A total of 49 US short-term acute-care hospitals.

**Patients:**

Patients hospitalized with CRE isolated from clinical cultures, April, 30, 2016, through August 31, 2017.

**Methods:**

We described characteristics of patients in CRACKLE-2 with nonintestinal CRE colonization and assessed the impact of site of colonization on clinical outcomes. We then compared outcomes of patients defined as having nonintestinal CRE colonization to all those defined as having infection. The primary outcome was a desirability of outcome ranking (DOOR) at 30 days. Secondary outcomes were 30-day mortality and 90-day readmission.

**Results:**

Of 547 patients with nonintestinal CRE colonization, 275 (50%) were from the urinary tract, 201 (37%) were from the respiratory tract, and 71 (13%) were from a wound. Patients with urinary tract colonization were more likely to have a more desirable clinical outcome at 30 days than those with respiratory tract colonization, with a DOOR probability of better outcome of 61% (95% confidence interval [CI], 53%–71%). When compared to 255 patients with CRE infection, patients with CRE colonization had a similar overall clinical outcome, as well as 30-day mortality and 90-day readmission rates when analyzed in aggregate or by culture site. Sensitivity analyses demonstrated similar results using different definitions of infection.

**Conclusions:**

Patients with nonintestinal CRE colonization had outcomes similar to those with CRE infection. Clinical outcomes may be influenced more by culture site than classification as “colonized” or “infected.”

The US Centers for Disease Control and Prevention (CDC) considers carbapenem-resistant Enterobacterales (CRE) an urgent public health challenge in both the 2013 and 2019 Antibiotic Resistance Threat Reports.^1,2^ Patients with invasive CRE infections have limited treatment options, frequent hospital readmissions, and high mortality rates.^3–5^ One study found similar in-hospital mortality with CRE pneumonia compared to bacteremia, highlighting the need to study nonbloodstream infections.^6^

Patients with CRE are often chronically ill, with frequent healthcare exposures, and they may have nonsterile site (ie, urinary tract, respiratory tract, or wound) cultures obtained for many reasons.^4,7^ Determining which CRE isolates obtained from cultures other than blood represent colonization and which represent infection requiring antimicrobial therapy, especially in seriously ill patients, poses a significant clinical challenge.^7^ Often, patients with CRE identified from clinical cultures but who do not meet criteria for an infection (considered colonization) are excluded from clinical studies, so little is known about the outcomes of these patients.^4,8–10^ Prior studies addressing CRE colonization have focused on patients with intestinal colonization identified through perirectal and/or rectal surveillance cultures and not on patients with CRE identified from clinical cultures.^11–13^

In this study, we have described the epidemiology and clinical outcomes of patients with nonintestinal CRE colonization identified in clinical cultures and compare differences by anatomic site of colonization. We also compared the clinical outcomes of patients defined as having CRE colonization to those defined as having CRE infection.

## Methods

### Study design, patient population, and inclusion criteria

CRACKLE-2 is a previously described prospective, observational cohort of patients with CRE from 49 US hospitals, enrolled from April 30, 2016, to August 31, 2017.^4^ We performed a secondary analysis of patients in CRACKLE-2 with CRE identified from clinical (not surveillance) cultures at colonization-eligible sites (urinary tract, respiratory tract, or wound). Trained staff abstracted patient demographics, clinical characteristics, and outcome data from the electronic health record (EHR).

Inclusion of CRE cases was based on antimicrobial susceptibility testing at local laboratories, according to the 2015 CDC definition, which included Enterobacterales that were resistant to carbapenems (minimum inhibitory concentration [MIC] ≥4 μg/mL for doripenem, meropenem, or imipenem or ≥2 μg/mL for ertapenem), that contained a carbapenemase gene, or that showed evidence of carbapenemase production. For Enterobacterales intrinsically resistant to imipenem, resistance to another nonimipenem carbapenem was required.^14^ Characterization of carbapenemase production was based on subsequent testing performed at central laboratories.^4^

CRACKLE-2 was approved by the institutional review boards of participating health systems with a waiver of consent.

### Definitions of infection, colonization, and antibiotic use

For the primary analysis, we used the CRACKLE-2 definition of infection in which study personnel classified infections using standardized definitions based on published criteria.^4^ The respiratory infection definition was based on American Thoracic Society and Infectious Diseases Society of America guidelines, and it included cultures from expectorated sputum, tracheal aspirates, bronchoalveolar lavage (BAL) or mini-BAL. Urinary tract and surgical site infection definitions were adapted from the CDC National Healthcare Safety Network surveillance criteria.^15–17^ For nonsurgical wounds, the definition required evidence of systemic inflammation on the day of culture and documentation of infection from the treating physician in EHR (Supplementary Table 1 online). Participants were classified based on the first CRE isolate obtained, and those who did not meet the definition of infection were classified as cases of colonization for the primary analysis. Due to significant heterogeneity and small sample size, patients with a culture from a site classified as “other” were excluded.

**Table 1.** Patient Demographics and Characteristics Stratified by Anatomic Culture Site and Infection Status

Variable	Urine		Respiratory		Wound		Total	
	Colonization (n = 275)^a^	Infection (n = 129)^a^	Colonization (n = 201)^a^	Infection (n = 67)^a^	Colonization (n = 71)^a^	Infection (n = 59)^a^	Colonization (n = 547)^a^	Infection (n = 255)^b^
**Region**^**c**^								
Midwest	50 (18)	28 (22)	36 (18)	8 (12)	14 (20)	5 (8)	100 (18)	41 (16)
Northeast	119 (43)	60 (47)	88 (44)	38 (57)	29 (41)	22 (37)	236 (43)	120 (47)
South	85 (31)	32 (25)	53 (26)	11 (16)	24 (34)	30 (51)	162 (30)	73 (29)
West	21 (8)	9 (7)	24 (12)	10 (15)	4 (6)	2 (3)	49 (9)	21 (8)
Age, median y (IQR)	68 (57–78)	62 (54–76)^d^	61 (55–72)	63 (54–75)	58 (50–71)	62 (51–69)	65 (55–76)^e^	62 (52–74)
Sex, male	120 (44)	60 (47)	129 (64)	47 (70)	44 (62)	41 (69)	293 (54)^e^	148 (58)
**Race**								
Black	96 (35)	42 (33)	55 (27)	18 (27)	26 (37)	23 (39)	177 (32)	83 (33)
White	130 (47)	54 (42)	97 (48)	33 (49)	33 (46)	25 (42)	260 (48)	112 (44)
Other	49 (18)	33 (26)	49 (24)	16 (24)	12 (17)	11 (19)	110 (20)	60 (24)
Hispanic ethnicity	31 (11)	21 (16)	22 (11)	12 (18)	10 (14)	6 (10)	63 (12)	39 (15)
Charlson comorbidity index >3	119 (43)	47 (36)	68 (34)	27 (40)	37 (52)	27 (46)	224 (41)	101 (40)
Diabetes	108 (39)	56 (43)	73 (36)	26 (39)	40 (56)	38 (64)	221 (40)^e^	120 (47)
**Admitted from**								
Home	163 (59)	75 (58)	106 (53)	30 (45)	39 (55)	40 (68)	308 (56)	145 (57)
Long-term chronic care facility	67 (24)	36 (28)	43 (21)	19 (28)	17 (24)	10 (17)	127 (23)	65 (25)
Long-term acute care facility	10 (4)	3 (2)	13 (6)	4 (6)	8 (11)	2 (3)	31 (6)	9 (4)
Transferred from another hospital or non-US country	35 (13)	15 (12)	39 (19)	14 (21)	7 (10)	7 (12)	81 (15)	36 (14)
Pitt bacteremia score ≥4	65 (24)	28 (22)	156 (78)	56 (84)	26 (37)	16 (27)	247 (45)^e^	100 (39)
In ICU at time of first CRE culture	44 (16)	13 (10)	124 (62)	46 (69)	21 (30)	15 (25)	189 (35)^e^	74 (29)
Hospital onset^f^	100 (36)	48 (37)	165 (82)	57 (85)	47 (66)	40 (68)	312 (57)^e^	145 (57)
No. of days from admission to CRE culture, median (IQR)	1 (0–6)	1 (0–10)	13 (3–30)	17 (5–33)	3 (1–15)	3 (1–16)	3 (0–17)^e^	3 (0–19)
**Organism**^e^								
*Klebsiella pneumoniae*	178 (65)	81 (63)	111 (55)	38 (57)	41 (58)	30 (51)	330 (60)	149 (58)
*Escherichia coli*	33 (12)	15 (12)	16 (8)	4 (6)	7 (10)	7 (12)	56 (10)	26 (10)
*Enterobacter* species	47 (17)	19 (15)	37 (18)	13 (19)	14 (20)	7 (12)	98 (18)	39 (15)
Non-*K. pneumoniae Klebsiella* spp	5 (2)	9 (7)	19 (9)	6 (9)	3 (4)	3 (5)	27 (5)	18 (7)
Other	12 (4)	5 (4)	18 (9)	6 (9)	6 (8)	12 (20)	36 (7)	23 (9)
**Isolate characterization**								
Carbapenemase-producing	175 (64)	84 (65)	129 (64)	41 (61)	41 (58)	32 (54)	345 (63)	157 (62)
Non–carbapenemase- producing	39 (14)	20 (16)	29 (14)	15 (22)	12 (17)	10 (17)	80 (15)	45 (18)
Unconfirmed CRE	61 (22)	25 (19)	43 (21)	11 (16)	18 (25)	17 (29)	122 (22)	53 (21)
**Received antibiotics**								
Empiric antibiotics^g^	166 (61)	100 (79)	161 (81)	59 (89)	53 (77)	53 (90)	380 (70)^e^	212 (84)^d^
Effective definitive antibiotics^h^	76 (28)	59 (48)	88 (48)	43 (69)	30 (45)	28 (50)	194 (38)^e^	130 (54)^d^

Note. IQR, interquartile range; CCI, Charlson comorbidity index; CRE, carbapenem-resistant Enterobacterales; ICU, intensive care unit.

a Data are no (%) unless otherwise stated.

b Only includes patients with CRE infections at colonization eligible sites (ie, urinary tract, respiratory tract or wound).

c Based on the US Census Bureau definitions.

d Indicates significant differences between colonization and infection categories at each anatomic culture site by χ^2^ or Kruskal-Wallis test (*P* < .05).

e Indicates significant differences between sites of colonization (urinary tract, respiratory tract, and wound) by χ^2^ or Kruskal-Wallis test (*P* < .05).

f Culture obtained >2 days after admission.

g Antibiotics received before antibiotic susceptibility results were available.

h Antibiotics received with 10 days of culture, after the susceptibility results were available with *in vitro* activity against the CRE isolate.

For a sensitivity analysis, we created a “clinician determined” definition based on whether clinicians documented infection or colonization in the EHR (Supplementary Table 1 online). Patients who were not classified as infected or colonized by EHR documentation were excluded from analysis using this definition (n = 172). Patients who met the definition of colonization by both CRACKLE-2 and the clinician-determined definition were termed “definitive colonization,” and those who met the definition of infection by both CRACKLE-2 and clinician-determined definitions were “definitive infections.” Patients who only met 1 definition of infection, and therefore 1 definition of colonization, were termed “possible infection/colonization.”

We have also reported, in aggregate, the proportion receiving empiric or definitive active antibiotic therapy with 1 of the following 8 gram-negative antibiotics or antibiotic classes: aminoglycosides, carbapenems, cefepime, ceftazidime-avibactam, fluoroquinolones, piperacillin-tazobactam, polymyxins and tigecycline. Empiric use was defined as administration after isolate collection and prior to antibiotic susceptibility results being available. Definitive, effective antibiotic therapy was defined as administration of an antibiotic within the first 10 days that was active against the isolate based on susceptibility testing and given after susceptibility results were available.

### Clinical outcomes

We used the same primary outcome as CRACKLE-2, a desirability of outcome ranking (DOOR) analysis that involves deriving a composite outcome, which incorporates counting the number of undesirable events that may occur throughout a patient’s clinical course.^4^ The DOOR methodology uses ordinal ranking to measure the global desirability of a patient outcome, combining benefits and risks.^18^ As in CRACKLE-2, we counted the following undesirable events that occurred within 30 days after the first qualifying CRE culture, including (1) unsuccessful discharge (remained hospitalized or readmitted within 30 days), (2) adverse events (eg, *Clostridioides difficile* infection and/or postculture renal failure), and (3) persistent symptoms or recurrence (ie, no symptomatic improvement, ongoing treatment with CRE-active antibiotics or recurrence of the same species of CRE at the same anatomic site).^4^ Patients alive without any of these events at 30 days were considered to have the most desirable outcome, and patients who died within 30 days, the least desirable. Between these 2 extremes, the 3 remaining ranks were alive with 1, 2, or 3 events. We also separately analyzed 30-day all-cause mortality and 90-day readmissions.

### Statistical analysis

We compared differences between site of culture in patients colonized with CRE and differences between CRE colonization and infection using the Kruskal-Wallis test for continuous variables and the Pearson χ^2^ for categorical variables. For the primary outcome, pairwise DOOR analyses were performed which estimate the probability that a randomly selected patient from one group will have a more desirable outcome than a randomly selected patient from another group.^18^ A probability near 50% with a 95% confidence interval (CI) that includes 50% indicates similar distributions of composite outcomes between groups. Because few patients had 3 undesirable events using DOOR, these patients were combined with the patients that had 2 undesirable events, as was done in the primary CRACKLE-2 analysis. We used inverse probability weighting (IPW) to adjust the DOOR probability and 30-day mortality for potential confounders. For the comparison of clinical outcomes based on anatomic site of CRE colonization (dependent variable) weights were calculated using the patient’s age, Charlson comorbidity index (CCI) (≤3 vs 3), patient’s location prior to admission (home vs other location), and Pitt bacteremia score. For the comparison of clinical outcomes based on infection versus colonization status (dependent variable) we removed the Pitt bacteremia score (which is associated with increased mortality even in the absence of bloodstream infections)^19^ as a confounder because we believed this was on the causal pathway between infection and clinical outcome. IPW resulted in balanced groups for the weighted variables. We did not perform sample size calculations as this was a secondary analysis of a completed cohort with a fixed sample size.

In a sensitivity analysis, we evaluated IPW-adjusted DOOR outcomes comparing colonization versus infection based on different definitions of infection (Supplementary Table 1 online). *P* < .05 were considered statistically significant. All analyses were performed in SAS version 9.4 software (SAS Institute, Cary, NC).

## Results

### Overall cohort

Of the 846 CRACKLE-2 patients eligible for inclusion, 547 (65%) were classified as having colonization and 255 (30%) as having an infection at colonization-eligible sites (urinary tract, respiratory tract, or wound) based on the CRACKLE-2 definition. Also, 44 (5%) were classified as having colonization from a site classified as “other” and were subsequently excluded due to significant heterogeneity (Supplementary Table 2 online includes characteristics and outcomes of this group).

**Table 2.** Desirability of Outcomes Ranking (DOOR), Mortality and Readmissions for Patients with Nonintestinal CRE Colonization Stratified by Anatomical Culture Site

Variable	Urinary Colonization (n = 2,750)^a^	Respiratory Colonization (n = 201)^a^	Wound Colonization (n = 71)^a^	Total (n = 547)^a^	*P* Value^b^
**DOOR at 30 d**					
Alive without events	154 (56)	61 (30)	28 (39)	243 (44)	N/A^c^
Alive with 1 event	77 (28)	22 (11)	21 (30)	120 (22)	
Alive with 2 or 3 events	11 (4)	57 (28)	13 (18)	81 (15)	
Dead	33 (12)	61 (30)	9 (13)	103 (19)	
**DOOR events at 30 days**^**d**^					
Remained hospitalized or readmitted within 30-d	59 (24)	63 (45)	23 (37)	145 (33)	<.01
Adverse event (renal failure or *Clostridioides difficile*)	15 (6)	8 (6)	7 (11)	30 (7)	.30
Persistent symptoms or recurrence^e^	25 (10)	65 (46)	19 (31)	109 (25)	<.01
30-d mortality	33 (12)	61 (30)	9 (13)	103 (19)	<.01
90-d readmissions^d^	111 (45)	38 (30)	26 (44)	175 (41)	.02

Note. CRE, carbapenem-resistant Enterobacterales; N/A, Not applicable.

a Data are no (%) unless otherwise stated.

b Comparing differences in urinary, respiratory and wound colonization with χ^2^ test.

c IPW-adjusted DOOR probability of a better outcome comparing urinary colonization versus respiratory colonization: 61% (95% CI, 53%–71%), urinary colonization versus wound colonization: 55% (95% CI, 47%–65%), and wound versus respiratory colonization: 57% (95% CI, 48%–67%).

d Denominator only includes patients who were discharged alive.

e No symptomatic improvement or ongoing treatment with CRE-active antibiotics or recurrence of the same species of CRE at the same anatomic site.

### Characterization of patients with nonintestinal CRE Colonization

CRE colonization occurred at the following included sites: 275 (50%) from the urinary tract, 201 (37%) from the respiratory tract, and 71 (13%) from a wound. More than half (n = 293, 54%) of the patients with CRE colonization were male and the median age was 65 years (interquartile range [IQR], 55–76). Also, 224 patients (41%) had a Charlson comorbidity index (CCI) >3. More than half of patients (n = 312, 57%) had a positive CRE culture identified >2 days after hospitalization, and 247 (45%) had a Pitt bacteremia score ≥4 on the day of culture (Table 1).

Patients with CRE urinary colonization compared to respiratory and wound colonization were more likely to be female (56% for urine vs 36% for respiratory vs 38% for wound; *P* < .01 for pairwise comparisons) and older median age (68 years [IQR, 57–78] for urine vs 61 years [IQR, 55–72] for respiratory vs 58 years [IQR, 50–71] for wound; *P* < .01 for pairwise comparisons). Patients with respiratory colonization were more likely to have a Pitt bacteremia score ≥4 (24% for urine vs 78% for respiratory vs 37% for wound; *P* < .01 for pairwise comparisons) and to have been admitted to the intensive care unit (ICU) at the time of culture (16% for urine vs 62% for respiratory vs 30% for wound; *P* < .01 for pairwise comparisons). More than half of the patients with CRE wound colonization had diabetes (n = 40, 56%), significantly higher than those with urinary (n = 108, 39%; *P* = .01) or respiratory colonization (n = 73, 36%; *P* < .01) (Table 1).

### Outcomes of patients with nonintestinal CRE colonization based on site of colonization

As shown in Table 2, 30 days after the index CRE culture, 243 (44%) patients with CRE colonization were alive without any undesirable DOOR events, 120 (22%) were alive with one event, 81 (15%) were alive with 2 or 3 events and 103 (19%) had died. For the primary IPW-adjusted DOOR analysis, patients with urinary colonization had a 61% chance (95% CI, 53%–71%) of having a more desirable outcome than patients with respiratory colonization after adjusting for age, CCI, location of patient prior to the hospitalization, and Pitt bacteremia score. Significant differences were not observed in the distribution of IPW-adjusted DOOR comparing urinary to wound colonization (DOOR probability estimate of a better outcome, 55%; 95% CI, 47%–65%) or wound to respiratory colonization (DOOR probability estimate of a better outcome, 57%; 95% CI, 48%–67%) (Table 2).

Patients with urinary or wound colonization demonstrated significantly lower IPW-adjusted 30-day mortality rates than those with respiratory colonization (urine vs respiratory adjusted difference: −8% (95% CI, −13% to −3%) and wound vs respiratory adjusted difference −12% (95% CI, −16% to −7%). The adjusted 30-day mortality was similar between those with urinary and wound colonization. Among patients discharged alive, patients with respiratory colonization had the lowest rate of 90-day readmissions (30%) (Table 2).

### Comparing CRE colonization versus CRE infection

CRE infection based on the CRACKLE-2 definition occurred at the following colonization-eligible sites: 129 (51%) from the urinary tract, 67 (26%) from the respiratory tract, and 59 (23%) from a wound (Table 1). Significant differences in demographics or clinical characteristics were not evident when comparing patients defined as having CRE infection to those defined as colonization, combining all anatomic sites. However, those with CRE infection were more likely to receive empiric (84% vs 70%, *P* < .001) and effective, definitive (54% vs 38%; *P* < .001) antibiotics with one of the previously listed 8 gram-negative antibiotics and/or antibiotic classes.

When comparing all patients with CRE colonization to those with CRE infection at colonization-eligible sites based on the CRACKLE-2 definition, differences in the IPW-adjusted DOOR outcome at 30 days were not seen (DOOR probability estimate of a better outcome, 49%; 95% CI, 45%–53%). Also, 30-day mortality was also similar comparing those infected to those colonized (17% vs 19%; IPW-adjusted difference, −2%; 95% CI, −11% to 8%) as was 90-day readmissions (48% vs 41%; *P* = .09) (Table 3).

**Table 3.** Desirability of Outcomes Ranking (DOOR), Mortality and Readmissions for Patients with Non-Intestinal CRE Colonization Compared to those with CRE Infection at Non-Sterile Culture Sites

Variable	CRE Colonization (n = 547)^a^	CRE Infection^b^ (n = 255)^a^	Total (n = 802)	*P* Value^c^
**DOOR at 30 d**				N/A^d^
Alive without events	243 (44)	116 (45)	359 (45)	
Alive with 1 event	120 (22)	57 (22)	177 (22)	
Alive with 2 or 3 events	81 (15)	39 (15)	120 (15)	
Dead	103 (19)	43 (17)	146 (18)	
**DOOR components at 30 d**^**e**^				
Remained hospitalized for readmitted within 30-d	145 (33)	81 (38)	226 (34)	0.16
Adverse event (renal failure or *Clostridioides difficile*)	30 (7)	8 (4)	38 (6)	0.12
Persistent symptoms or recurrence^f^	109 (25)	46 (22)	155 (24)	0.42
30-day mortality	103 (19)	43 (17)	146 (18)	0.50
90-day readmissions^e^	175 (41)	99 (48)	274 (43)	0.09

Note. CRE, carbapenem-resistant Enterobacterales; IPW, inverse probability weighting.

a Data are no (%) unless otherwise stated.

b Only includes patients with CRE infections at colonization-eligible anatomic sites (ie, urinary tract, respiratory tract, and wound sources).

c Comparing differences in colonization versus infection with χ^2^ test.

d IPW-adjusted DOOR probability of a better outcome comparing colonization to infection: 49% (95% CI, 45%–53%).

e Denominator only includes patients who were discharged alive.

f No symptomatic improvement or ongoing treatment with CRE-active antibiotics or recurrence of the same species of CRE at the same anatomic site.

**Fig. 1.**
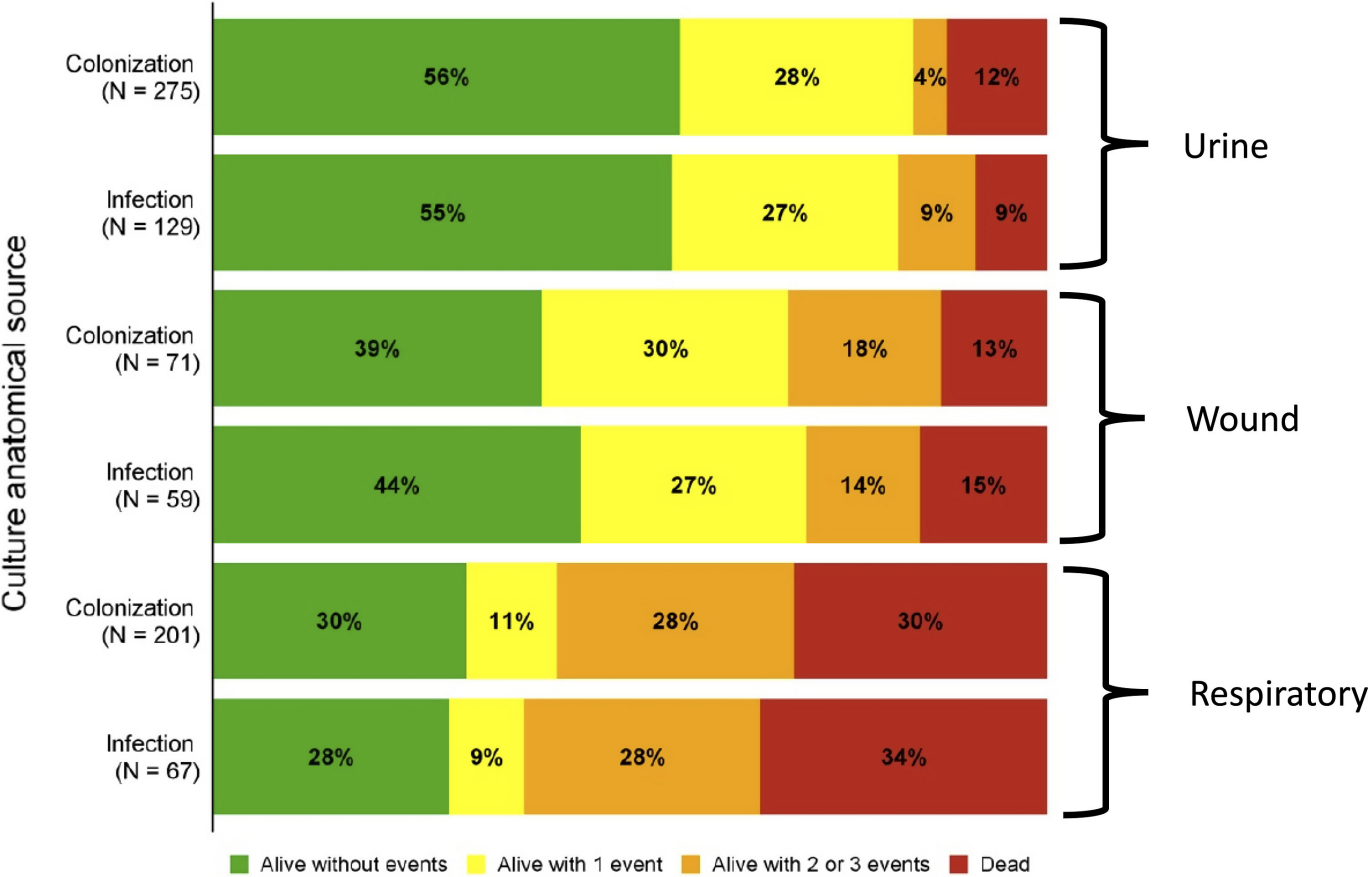
Unadjusted desirability of outcomes ranking (DOOR) comparing infection versus colonization by anatomic culture site.

We did not detect differences in the distribution of IPW-adjusted DOOR outcomes comparing colonized versus infected patients, stratified by culture site (Fig. 1 and Supplementary Table 3 online). Similarly, we did not detect differences in the secondary outcomes of adjusted 30-day mortality and 90-day readmissions, stratified by anatomic culture site (Supplementary Table 3 online).

In a sensitivity analysis using the clinician-determined definition of infection (Supplementary Table 1 online), we detected no significant differences seen in the distribution of IPW-adjusted DOOR outcomes comparing CRE colonization and infection (DOOR probability estimate of a better outcome, 52%; 95% CI, 47–57%) (Supplementary Table 4 online). Additionally, when we compared patients who met the definition of CRE colonization by both the CRACKLE-2 and the clinician-determined definition (“definitive colonization”) to those who met the definition of infection by both definitions (“definitive infection”), differences in DOOR outcomes were not seen (DOOR probability estimate of a better outcome with colonization: 52%, 95% CI, 46%–59%) (Supplementary Table 5 online).

## Discussion

In this analysis of ∼800 hospitalized individuals across the United States, patients with nonintestinal CRE colonization demonstrated similar clinical outcomes to those with CRE infections of the same anatomic culture site. Among patients with nonintestinal CRE colonization, those with respiratory tract colonization had the worst overall clinical outcomes using DOOR, including the highest crude 30-day mortality rate (30%). Our results are similar to prior studies demonstrating that patients with respiratory infections caused by multidrug-resistant gram-negative organisms have high all-cause mortality rates (∼25%–40%) and experience worse outcomes than patients with infections at other nonsterile sites.^6,20,21^ To our knowledge, we are the first to specifically compare outcomes of patients colonized to those infected with CRE in the respiratory tract. The observed high mortality (30%) in both groups suggests that underlying comorbidities may contribute more to mortality than the CRE infection in the respiratory tract.

This study is unique because we did not assess intestinal CRE colonization and instead focused on a more common scenario of CRE isolated in clinical cultures but deemed not to represent infection. Unexpectedly, we found that patients determined to have CRE colonization experienced similarly poor outcomes as patients determined to have CRE infection. These results persisted when analyzed by anatomic site and when independently analyzed for 30-day mortality or 90-day readmission rates. These results suggest that patients who have CRE cultured from any site, whether actively infected or not, are often chronically ill, frequently admitted to healthcare facilities, and overall, at high risk for mortality and other poor outcomes. In one study looking at nearly 20 years of carbapenem-resistant gram-negative isolates (colonization and infection) from a single institution, there were high rates of comorbidities and 41% of the patients were in the ICU at the time of culture. The 30-day mortality rate was similar to our study (19%) and the overall mortality rate at 1 year was 47%.^22^

The lack of difference in outcomes between colonization and infection may in part be explained by the difficulty clinicians have in differentiating colonization from infection. Large-scale surveillance studies are also frequently not able to determine which clinical cultures represent true infections.^7,23^ In CRACKLE-2, the definition of infection was based on standardized criteria extracted from the EHR. However, when we also applied a definition of infection that was determined based on physician documentation, our results did not change. Even using the strictest definitions of infection and colonization (ie, patients that met both the CRACKLE-2 and the clinician-determined definition for infection or colonization), we still did not observe differences in outcomes. Notably, the infected group in this study only included patients with CRE isolated from a colonization-eligible site (eg, urinary tract, respiratory tract, or wound) and did not include those with CRE in a normally sterile site (ie, blood), which is usually associated with increased mortality.^7,24^ In the initial CRACKLE-2 study where patients with infections at normally sterile sites were included, the all-cause 30-day mortality was 24% which was higher than the 17% we observed.^4^

In this population, 70% of colonized patients received empiric antibiotics, indicating substantial overlap of antibiotic use between those colonized and infected. However, a much smaller percentage (38%) of patients with CRE colonization received effective, definitive antibiotics. Our results are representative of “real-world” practices where frequent antibiotic changes are made and it is often necessary to give antibiotics before determining when a patient is truly infected.^21^ Additional studies are needed to define when to use antibiotics in patients with CRE isolated from clinical cultures, and if antibiotic use influences clinical outcomes in scenarios in which it is difficult to determine whether the patient is infected.

The strengths of this study include a large sample of CRE isolates from many centers across the United States. We systematically applied standardized definitions of infection or colonization. We also primarily utilized a DOOR outcome, which can better capture the global desirability of a patient’s experience instead of solely looking at 30-day mortality.^8,25^

The study also had several limitations. CRACKLE-2 study sites were predominantly large, academic hospitals, which may limit the generalizability to smaller hospitals with different patient populations and culture utilization practices. We did not collect data on the reason for culture collection, which may have been informative for patients determined to not meet the standardized infection criteria. Determination of clinical diagnosis and infection status were conducted retrospectively and relied on EHR documentation. Lastly, we used the same primary DOOR outcome that was used in the CRACKLE-2 study to allow standardization across studies and generalizability. However, some events in the DOOR framework, such as persistent symptoms, can be difficult to interpret in the setting of colonization. These events were evaluated independently from the classification of infection versus colonization, and because the DOOR analysis ultimately aims to encompass the full patient experience, we thought it was important to keep persistent symptoms and recurrence included.

In summary, patients with CRE cultured from the respiratory tract are likely to have worse clinical outcomes than patients with CRE cultured from the urinary tract or a wound, with a 30-day mortality of >30% even without objective criteria for infection. Additionally, patients who are deemed to have CRE colonization may do as poorly as patients with CRE infection at any, nonsterile culture site. Future work should focus on how to effectively differentiate infection versus colonization and determine if certain characteristics of colonized patients, including the number of colonized sites, can help predict who benefits from antibiotics and should be included in future clinical studies.

## Supplementary material

For supplementary material accompanying this paper visit http://doi.org/10.1017/ice.2022.4. click here to view supplementary material
