# Supplementary material for: Poor outcomes in both infection and colonization with carbapenem-resistant Enterobacterales
Source: Infect Control Hosp Epidemiol. 2022 Feb 2;43(12):1840–6. doi: 10.1017/ice.2022.4 (PMC9343470; doi:10.1017/ice.2022.4)
Supplement: Supplementary file 1 [file S0899823X22000046sup001.docx]

**Supplemental Table 1**: Definitions of CRE Infection (CRACKLE-2 versus Clinician-determined) Stratified by Culture Site

|  | Respiratory | Urine | Wound |
| --- | --- | --- | --- |
| CRACKLE-2 infection | 1. CRE cultured at any time during admission with:   - New lobar or multilobar infiltrate(s) OR interstitial infiltrate(s) on chest x-ray   AND   - At least one of the following:   - Maximum temperature >38°C   - Purulent or non-purulent sputum production   OR  2. CRE cultured >2 days after hospital admission with:   - New lobar or multilobar infiltrate(s) AND interstitial infiltrate(s) on chest x-ray are not present   AND   - All of the following:   - Maximum temperature > 38°C   - Purulent sputum production   - Highest WBC > 10 (10^3^/mcl) | 1. CRE cultured with at least one of the following:  - Dysuria - Urinary frequency - Urinary urgency - Flank pain - Pelvic supra-pubic pain - Maximum temperature > 38°C if catheter associated - Maximum temperature > 38°C and ≤65 years old if non-catheter associated   OR   1. An additional CRE blood culture with the same species as the urine culture isolated within 3 days after the urine culture. | 1. CRE cultured from a surgical/post-surgical wound (superficial/subcutaneous tissue) with ≥1 of the following:   - - Maximum temperature > 38°C   - Surgical intervention   OR  2. CRE cultured from a surgical wound (deep soft tissue or bone) with ≥1 of the following:   - - Purulent drainage from wound   - Surgical intervention for this wound and either maximum temperature >38°C or localized pain   OR  3. CRE cultured from a non-surgical wound   - Documented clinical diagnosis of wound infection and ≥1 of the following:   - Highest WBC >10 (10^3^/mcl)   - Highest WBC< 4 (10^3^/mcl)   - Maximum temperature >37.5°C or <35.5°C |
| CRACKLE-2 colonization | - Patients not meeting the definition for infection | - Patients not meeting the definition for infection | - Patients not meeting the definition for infection |
| Clinician-determined infection^1^ | - Tracheobronchitis or pneumonia (ventilated or non-ventilated) documented in the EHR | - Cystitis, pyelonephritis, prostatitis or UTI not otherwise defined documented in the EHR | - Wound infection documented in EHR |
| Clinician-determined colonization^1^ | - Respiratory colonization documented in EHR^2^ | - Urinary colonization documented in the EHR^2^ | - Wound colonization documented in the EHR^2^ |

1. Determined based off infectious diseases clinical notes if available. If not available, the primary team clinical notes were used

2. Patients classified as unknown or “other” by the data entry team were excluded

Abbreviations: CRE, carbapenem-resistant Enterobacterales; EHR, electronic health record; UTI, urinary tract infection; WBC, white blood cell count

**Supplemental Table 2:** Demographics, Characteristics, and Outcomes of Patients with CRE Colonization Identified in Culture Sites Other than the Urinary Tract, Respiratory Tract or Wound

|  | Other colonization  (n = 44) |
| --- | --- |
| **Region^1^** |  |
| Midwest | 6 (14) |
| Northeast | 13 (30) |
| South | 19 (43) |
| West | 6 (14) |
| **Age in years, median (IQR)** | 64 (54, 76) |
| **Male** | 28 (64) |
| **Race** |  |
| Black | 19 (43) |
| White | 22 (50) |
| Other | 3 (7) |
| **Hispanic ethnicity** | 0 (0) |
| **CCI >3** | 16 (36%) |
| **Diabetes** | 16 (36) |
| **Admitted from** |  |
| Home | 27 (61) |
| Long term chronic care facility | 6 (14) |
| Long term acute care facility | 2 (5) |
| Transferred from another hospital or Non-U.S. country | 9 (20) |
| **Pitt bacteremia score ≥4** | 13 (30) |
| **In ICU at time of first CRE culture** | 11 (25) |
| **Hospital onset^2^** | 27 (61) |
| **Number of days from admission to CRE culture, median (IQR)** | 3 (0, 12.5) |
| **Organism** |  |
| *Klebsiella pneumoniae* | 25 (57) |
| *Escherichia coli* | 6 (14) |
| *Enterobacter* species | 9 (20) |
| Non-*K. pneumoniae Klebsiella* species | 1 (2) |
| Other | 3 (7) |
| **Isolate characterization** |  |
| Carbapenemase-producing | 20 (45) |
| Non-carbapenemase-producing | 12 (27) |
| Unconfirmed CRE | 12 (27) |
| **Received antibiotics** |  |
| Empiric antibiotics^3^ | 35 (80) |
| Effective definitive antibiotics^4^ | 16 (41) |
| **DOOR at 30 days** |  |
| Alive without events | 17 (39) |
| Alive with 1 event | 11 (25) |
| Alive with 2 or 3 events | 8 (18) |
| Dead | 8 (18) |
| **DOOR events at 30 days^5^** |  |
| Remained hospitalized or readmitted within 30-days | 13 (36) |
| Adverse event (renal failure or *Clostridioides difficile*) | 4 (11) |
| Persistent symptoms or recurrence^6^ | 10 (28) |
| **30-day mortality** | 8 (18) |
| **90-day readmissions^5^** | 11 (31) |

Data are n (%) unless otherwise stated

1. Based on the U.S. Census Bureau definitions
2. Culture obtained >2 days after admission
3. Antibiotics received before antibiotic susceptibility results were available.
4. Antibiotics received with 10 days of culture, after the susceptibility results were available with *in vitro* activity against the CRE isolate
5. Denominator only includes patients who were discharged alive
6. No symptomatic improvement or ongoing treatment with CRE-active antibiotics or recurrence of the same species of CRE at the same anatomic site

Abbreviations: IQR, interquartile range; CCI, Charlson comorbidity index; CRE, carbapenem-resistant Enterobacterales; ICU, intensive care unit

**Supplemental Table 3:** Desirability of Outcome Ranking (DOOR) for Patients with CRE Colonization and CRE Infection by Anatomic Culture Site

|  | **CRE Colonization** | **CRE Infection** | **Total** |
| --- | --- | --- | --- |
| ***Urinary tract cultures only*** | n = 275 | n = 129 | n = 404 |
| DOOR at 30 days^1^ |  |  |  |
| Alive without events | 154 (56) | 71 (55) | 225 (56) |
| Alive with 1 event | 77 (28) | 35 (27) | 112 (28) |
| Alive with 2 or 3 events | 11 (4) | 12 (9) | 23 (6) |
| Dead | 33 (12) | 11 (9) | 44 (11) |
| 90-day readmissions^2,3^ | 111 (45) | 58 (50) | 169 (47) |
| ***Respiratory tract cultures only*** | n = 201 | n = 67 | n = 268 |
| DOOR at 30 days^4^ |  |  |  |
| Alive without events | 61 (30%) | 19 (28%) | 80 (30%) |
| Alive with 1 event | 22 (11%) | 6 (9%) | 28 (10%) |
| Alive with 2 or 3 events | 57 (28%) | 19 (28%) | 76 (28%) |
| Dead | 61 (30%) | 23 (34%) | 84 (31%) |
| 90-day readmissions^2,3^ | 38 (30) | 15 (35) | 53 (31) |
| ***Wound cultures isolates only*** | n = 71 | n = 59 | n = 130 |
| DOOR at 30 days^5^ |  |  |  |
| Alive without events | 28 (39) | 26 (44) | 54 (42) |
| Alive with 1 event | 21 (30) | 16 (27) | 37 (28) |
| Alive with 2 or 3 events | 13 (18) | 8 (14) | 21 (16) |
| Dead | 9 (13) | 9 (15) | 18 (14) |
| 90-day readmissions^2,3^ | 26 (44) | 26 (53) | 52 (48) |

1. IPW-adjusted DOOR probability of a better outcome with CRE colonization versus infection: 51% (95% CI 45%–56)
2. Denominator only includes patients discharged alive
3. No significant difference comparing colonization versus infection by Chi-square test
4. IPW-adjusted DOOR probability of a better outcome with CRE colonization versus infection: 52% (95% CI 45%–60%)
5. IPW-adjusted DOOR probability of a better outcome with CRE colonization versus infection: 51% (95% CI 40%–62%)

Abbreviations: CRE, carbapenem-resistant Enterobacterales; IPW, inverse probability weighting

**Supplemental Table 4**: Desirability of Outcome Ranking (DOOR) for Patients with CRE Colonization and those with CRE Infection Based on the Clinician-Determined Definition

|  | **CRE Colonization**  **(n = 136)** | **CRE Infection**  **(n = 494)** | **Total^1^**  **(n = 630)** |
| --- | --- | --- | --- |
| DOOR at 30 days^2^ |  |  |  |
| Alive without events | 66 (49) | 233 (47) | 299 (47) |
| Alive with 1 event | 35 (26) | 103 (21) | 138 (22) |
| Alive with 2 or 3 events | 14 (10) | 72 (15) | 88 (14) |
| Dead | 21 (15) | 86 (17) | 107 (17) |

1. All culture sites, excludes those with a missing or unknown clinician-determined diagnosis

2. IPW-adjusted DOOR probability of a better outcome with CRE colonization versus infection: 52% (95% CI 47–57%)

Abbreviations: CRE, carbapenem-resistant Enterobacterales; IPW, inverse probability weighting

**Supplemental Table 5**: Desirability of Outcome Ranking (DOOR) for Patients with CRE Colonization and those with CRE Infection Based on a Combined Definition

|  | **Definitive Infection^1^**  **(n = 203)** | **Possible Infection/Colonization^2^**  **(n = 312)** | **Definitive Colonization^3^**  **(n = 115)** | **Total^4^**  **(n = 630)** |
| --- | --- | --- | --- | --- |
| **DOOR at 30 days^5^** |  |  |  |  |
| Alive without events | 95 (47) | 149 (48) | 55 (48) | 299 (47) |
| Alive with 1 event | 42 (21) | 66 (21) | 30 (26) | 138 (22) |
| Alive with 2 or 3 events | 33 (16) | 40 (13) | 13 (11) | 86 (14) |
| Dead | 33 (16) | 57 (18) | 17 (15) | 107 (17) |

1. Met both the CRACKLE-2 and clinician-determined definition of infection
2. Met either the CRACKLE-2 or clinician-determined definition of infection
3. Met both the CRACKLE-2 and clinician-determined definition of colonization
4. Excludes those with missing or unknown clinician-determined diagnosis
5. IPW-adjusted DOOR probability of a better outcome comparing definitive colonization to definitive infection: 52% (46%–59%); definitive colonization to possible infection/colonization: 52% (45%–58%); possible infection/colonization to definitive infection: 51% (46%–56%)

Abbreviations: CRE, carbapenem-resistant Enterobacterales; IPW, inverse probability weighting
